# Resposta da Pressão Arterial e Ecocardiografia sob Estresse Físico: Novas Perspectivas sobre um Desafio Contemporâneo

**DOI:** 10.36660/abc.20230737

**Published:** 2023-12-14

**Authors:** Eduardo M. Vilela, Ricardo Fontes-Carvalho

**Affiliations:** 1 Serviço de Cardiologia Centro Hospitalar de Vila Nova de Gaia/Espinho Vila Nova de Gaia Portugal Serviço de Cardiologia , Centro Hospitalar de Vila Nova de Gaia/Espinho , Vila Nova de Gaia – Portugal; 2 Centro de Investigação Cardiovascular Faculdade de Medicina Universidade do Porto Porto Portugal Centro de Investigação Cardiovascular , Faculdade de Medicina , Universidade do Porto , Porto – Portugal

**Keywords:** Pressão Arterial, Doença Arterial Coronariana, Ecocardiografia/métodos, Ecocardiografia sob Estresse/métodos, Ecocardiografia/tendências

O exercício impacta substancialmente o sistema cardiovascular e está associado a muitos benefícios potenciais em diferentes momentos do continuum cardiovascular. ^1 , 2^ Por outro lado, o exercício físico tem sido postulado há várias décadas como uma possível abordagem para avaliar a resposta cardiovascular. ^3^ Como tal, esta metodologia pode ser de interesse para fins de diagnóstico, ao mesmo tempo que fornece informações prognósticas. ^3^ Embora o amadurecimento de protocolos que abrangem a resposta eletrocardiográfica ao exercício tenha proporcionado grandes avanços, novas técnicas permitiram uma visão ampliada da complexa interação entre o exercício e o sistema cardiovascular. ^3 - 5^

A ecocardiografia sob estresse físico (EEF) tem evoluído progressivamente para um quadro de grande relevância, nomeadamente na avaliação da doença arterial coronariana (DAC). ^4^ A ecocardiografia também tem a vantagem de avaliar componentes como capacidade de exercício, função diastólica, gradientes dinâmicos e valvopatia. ^4 , 6^ Além disso, a pressão arterial (PA) e a monitorização eletrocardiográfica também fornecem informações essenciais. ^4^ Dados recentes também mostram o potencial da combinação com outras técnicas, como a ultrassonografia pulmonar, para avaliar a congestão. ^7^ Embora, conforme ilustrado nas diretrizes contemporâneas, a EEF tenha obtido grande interesse (sendo preferido aos testes farmacológicos em pacientes capazes de praticar exercício), ainda existem algumas ressalvas na sua aplicação ideal. ^4 , 8^

Incorporar a avaliação da PA durante o teste ergométrico é fundamental para fornecer uma perspectiva abrangente da resposta cardiovascular. ^4 , 9^ Durante o exercício, espera-se que a PA sistólica (PAS) aumente, embora aumentos acima de certos limites sejam considerados anormais [sendo definidos como uma resposta hipertensiva ao exercício (RHE)]. ^4 , 10^ Embora as reduções na PAS durante o exercício tenham sido descritas como potenciais precursores de risco aumentado, o impacto global de uma RHE tem sido um tema de discussão. ^3 , 11 , 12^ Embora os dados descrevam uma associação potencial entre uma RHE e eventos adversos, fatores como os pontos de corte utilizados, a carga de trabalho alcançada e a aptidão cardiorrespiratória devem ser considerados. ^8 , 12 , 13^ Além disso, embora dados anteriores sugerissem uma possível associação entre uma RHE durante a EEF e um resultado positivo na ausência de DAC significativa, alguns dados não apresentaram esta associação. ^4 , 8^ A este respeito, um estudo clássico não relatou diferenças em falsos positivos para indivíduos com valores normais em comparação com aqueles com elevações anormais na PAS. ^8^ Curiosamente, outro estudo abrangendo 21.949 pacientes (embora deva ser sublinhado que no contexto da ecocardiografia sob estresse com dobutamina) relatou que aqueles com RHE não tinham maior probabilidade de apresentar resultados falso-positivos, embora tivessem menor probabilidade de ter DAC de grau superior ou multiarterial. ^14^

Neste contexto, Martins-Santos et al., ^13^ fornecem insights derivados de um estudo interessante que visa fornecer dados sobre a relação entre uma RHE sistólico (RHES) durante a EEF e a isquemia (avaliada por alterações na contratilidade segmentar). ^13^ Também é fornecida uma descrição detalhada dos sintomas, alterações eletrocardiográficas e parâmetros da função diastólica. Este estudo abrangeu 14.367 indivíduos (52% do sexo feminino, com idade entre 58±11 anos) submetidos à EEF no contexto de síndromes coronarianas crônicas estabelecidas ou suspeitas, dos quais 10,4% apresentavam RHES (definido como aumento >90 mmHg, descrito como o percentil 95 na população em estudo). ^13^ Aqueles com RHES eram mais jovens, mais frequentemente do sexo masculino e tinham maior prevalência de hipertensão arterial basal e obesidade. Embora não tenha havido diferenças entre os grupos na história prévia de dor torácica atípica, aqueles que apresentavam RHES eram mais frequentemente assintomáticos antes do exame, enquanto aqueles que não apresentavam tinham maior probabilidade de ter tido dor torácica típica prévia. ^13^ Como esperado, aqueles com RHES alcançaram pico de PAS mais alto e níveis superiores de pico de duplo produto. Curiosamente, embora as alterações do segmento ST tenham sido mais frequentes neste grupo, a angina durante a EEF foi menos frequente. Além disso, as alterações isquêmicas (em relação à avaliação ecocardiográfica) foram significativamente menos frequentes neste grupo (81,9% com padrão de contratilidade segmentar normal vs 75,8% naqueles sem RHES). Na verdade, neste estudo, uma RHES foi inversamente associado ao desenvolvimento de isquemia. Notavelmente, apresentar angina durante o exame foi o mais forte preditor de isquemia. ^13^ Este estudo acrescenta informações relevantes à literatura sobre o tema, sendo o tamanho da amostra e a caracterização da população aspectos importantes. Entretanto, conforme reconhecido pelos autores, devem ser observadas limitações como o desenho do estudo e a exclusão de pacientes que não apresentavam aumento de PAS. Além disso, deve também ser considerada a falta de informação angiográfica ou de dados sobre eventos cardiovasculares (nomeadamente naqueles com alterações do segmento ST mas contratilidade segmentar normal).

A DAC continua a ser uma patologia desafiadora cujos princípios fundamentais estão em constante mudança à medida que novos dados, num contexto de técnicas mais avançadas e integrativas, permitem melhorias na compreensão desta entidade complexa. ^3 , 4 , 15^ À medida que se dá cada vez mais ênfase à relevância de uma abordagem abrangente da DAC, reavaliar o papel da resposta da PA e a sua interação com fatores auxiliares pode ser outro passo importante na jornada contínua para um cuidado individualizado e otimizado centrado no paciente.

## References

[1] Valenzuela PL, Ruilope LM, Santos-Lozano A, Wilhelm M, Kränkel N, Fiuza-Luces C (2023). Exercise benefits in cardiovascular diseases: from mechanisms to clinical implementation. Eur Heart J.

[2] Fontes-Carvalho R, Vilela EM, Gonçalves-Teixeira P, Watson RR, Zibadi S (2018). Lifestyle in heart health and disease.

[3] Fletcher GF, Ades PA, Kligfield P, Arena R, Balady GJ, Bittner VA (2013). Exercise standards for testing and training: a scientific statement from the American Heart Association. Circulation.

[4] Pellikka PA, Arruda-Olson A, Chaudhry FA, Chen MH, Marshall JE, Porter TR (2020). Guidelines for Performance, Interpretation, and Application of Stress Echocardiography in Ischemic Heart Disease: From the American Society of Echocardiography. J Am Soc Echocardiogr.

[5] Vilela EM, Fontes-Carvalho R (2023). Left Ventricular Mechanics: Untwisting the Pathways of the Cardiovascular Response to Exercise. Arq Bras Cardiol.

[6] Cotrim CA, Café H, João I, Cotrim N, Guardado J, Cordeiro P (2022). Exercise stress echocardiography: Where are we now?. World J Cardiol.

[7] Merli E, Ciampi Q, Scali MC, Zagatina A, Merlo PM, Arbucci R (2022). Pulmonary Congestion During Exercise Stress Echocardiography in Ischemic and Heart Failure Patients. Circ Cardiovasc Imaging.

[8] Jurrens TL, From AM, Kane GC, Mulvagh SL, Pellikka PA, McCully RB (2012). An exaggerated blood pressure response to treadmill exercise does not increase the likelihood that exercise echocardiograms are abnormal in men or women. J Am Soc Echocardiogr.

[9] Vilela EM, Ladeiras-Lopes R, João A, Torres S, Ribeiro J, Campos L (2021). Cardiac rehabilitation in elderly myocardial infarction survivors: focus on circulatory power. Rev Cardiovasc Med.

[10] Périard JD, Wilson MG, Drezner JA, Sharma S (2017). IOC Manual of Sports Cardiology.

[11] Hedman K, Lindow T, Cauwenberghs N, Carlén A, Elmberg V, Brudin L (2022). Peak exercise SBP and future risk of cardiovascular disease and mortality. J Hypertens.

[12] Nayor M, Gajjar P, Murthy VL, Miller PE, Velagaleti RS, Larson MG (2023). Blood Pressure Responses During Exercise: Physiological Correlates and Clinical Implications. Arterioscler Thromb Vasc Biol.

[13] Martins-Santos CB, Duarte LTA, Ferreira-Junior CR, Feitosa AGT, Oliveira EVG, Campos ICMB (2023). Exaggerated Systolic Blood Pressure Increase with Exercise and Myocardial Ischemia on Exercise Stress Echocardiography. Arq Bras Cardiol.

[14] Abram S, Arruda-Olson AM, Scott CG, Pellikka PA, Nkomo VT, Oh JK (2017). Frequency, Predictors, and Implications of Abnormal Blood Pressure Responses During Dobutamine Stress Echocardiography. Circ Cardiovasc Imaging.

[15] Marzlin N, Hays AG, Peters M, Kaminski A, Roemer S, O’Leary P (2023). Myocardial Work in Echocardiography. Circ Cardiovasc Imaging.

