# Supplementary material for: Resposta da Pressão Arterial e Ecocardiografia sob Estresse Físico: Novas Perspectivas sobre um Desafio Contemporâneo
Source: Arq Bras Cardiol. 2023 Dec 14;120(11):e20230737. [Article in Portuguese] doi: 10.36660/abc.20230737 (PMC10763685; doi:10.36660/abc.20230737)
Supplement: Translation

# Translation: Blood Pressure Response and Exercise Stress Echocardiography: New Perspectives on a Contemporary Challenge

**Authors:** Eduardo M. Vilela, Ricardo Fontes-Carvalho

**Affiliations:** 1 Portugal Serviço de Cardiologia, Centro Hospitalar de Vila Nova de Gaia/Espinho, Vila Nova de Gaia – Portugal; 2 Portugal Centro de Investigação Cardiovascular, Faculdade de Medicina, Universidade do Porto, Porto – Portugal

**Keywords:** Arterial Pressure, Coronary Artery Disease, Echocardiography/methods, Echocardiography, Stress/methods, Echocardiography/trends

Exercise substantially impacts the cardiovascular system and is associated with many potential benefits across different momentums of the cardiovascular continuum. ^1 , 2^ On the other hand, physical exercise has for several decades been postulated as a possible approach to assess the cardiovascular response. ^3^ As such, this methodology could be of interest for diagnostic purposes while also providing prognostic information. ^3^ While the maturation of protocols encompassing the electrocardiographic response to exercise provided major breakthroughs, novel techniques have allowed an expanded view of the complex interplay between exercise and the cardiovascular system. ^3 - 5^

Exercise stress echocardiography (ESE) has progressively evolved into a highly relevant framework, namely when assessing coronary artery disease (CAD). ^4^ Echocardiography also has the advantage of assessing components such as exercise capacity, diastolic function, dynamic gradients, and valvular heart disease. ^4 , 6^ Furthermore, blood pressure (BP) and electrocardiographic monitoring also provide pivotal inputs ^4^ . Recent data also showcases the potential of combining with other techniques, such as lung ultrasound, to assess congestion. ^7^ While, as illustrated in contemporary guidelines, ESE has garnered great interest (being preferred to pharmacological testing in patients able to exercise), there are still some caveats in its optimal application. ^4 , 8^

Incorporating BP evaluation during exercise testing is paramount to providing a comprehensive cardiovascular response outlook. ^4 , 9^ During exercise, systolic BP (SBP) is expected to increase, although increases above certain thresholds are considered abnormal [being defined as a hypertensive response to exercise (HRE)]. ^4 , 10^ While decreases in SBP during exercise have been described as potential harbingers of increased risk, the overall impact of a HRE has been a topic of discussion. ^3 , 11 , 12^ Although data describes a potential association between a HRE and adverse events, factors such as the cut-offs used, workload achieved, and cardiorespiratory fitness should be considered. ^8 , 12 , 13^ Furthermore, while prior data suggested a possible association between a HRE during ESE and a positive result in the absence of significant CAD, some data did not present this association. ^4 , 8^ In this regard, a classical study reported no differences in false positives for subjects with normal compared to those with abnormal elevations in SBP. ^8^ Interestingly, another study encompassing 21949 patients (albeit it should be underscored that in the setting of dobutamine stress echocardiography) reported that those with a HRE were not more likely to have false positive results, albeit being less likely to have higher-grade or multivessel CAD. ^14^

In this background, Martins-Santos et al. ^13^ provide insights derived from an interesting study aiming to provide data on the relationship between a systolic HRE (SHRE) during ESE and ischemia (assessed by changes in segmental contractility). ^13^ A detailed description of symptoms, electrocardiographic changes, and diastolic function parameters is also provided. This study encompassed 14367 individuals (52% female gender, aged 58±11 years old) who underwent ESE in the setting of established or suspected chronic coronary syndromes, of whom 10.4% had a SHRE (defined as an increase >90 mmHg, described as the 95 ^th^ percentile in the population under study). ^13^ Those with a SHRE were younger, more often male, and had a higher prevalence of baseline arterial hypertension and obesity. While there were no differences between groups in a prior history of atypical chest pain, those who presented a SHRE were more frequently asymptomatic prior to the exam, whereas those who did not were more likely to have had prior typical chest pain. ^13^ As expected, those with a SHRE achieved a higher peak SBP and superior levels of peak double-product. Interestingly, although ST-segment changes were more frequent in this group, angina during ESE was less frequent. Moreover, ischemic changes (regarding echocardiographic assessment) were significantly less frequent in this group (81.9% having a normal segmental contractility pattern *vs* 75.8% in those without a SHRE). Indeed, in this study, a SHRE was inversely associated with developing ischemia. Notably, presenting angina during the test was the strongest predictor of ischemia. ^13^ This study adds relevant information to the literature on this topic, with the sample size and population characterization being important aspects. However, as acknowledged by the authors, limitations such as the study design and the exclusion of patients who did not present increases in SBP should be noted. In addition, the lack of angiographical information or data on cardiovascular events (namely in those with ST-segment changes but normal segmental contractility) should also be considered.

CAD remains a challenging pathology whose core tenets are continuously shifting as new data, on a background of more advanced and integrative techniques, allows for improvements in understanding this complex entity. ^3 , 4 , 15^ As an increasing emphasis is placed on the relevance of a comprehensive approach to CAD, reappraising the role of the BP response and its interplay with ancillary factors may be another important step on the continuous journey to individualized and optimized patient-centered care.
